# A balancing act: a qualitative exploration of factors influencing how activities coordinators plan and facilitate music activities in Scottish care homes

**DOI:** 10.1002/alz70858_105699

**Published:** 2025-12-26

**Authors:** Anna Dalziel Bryan, Tom C Russ, Katie Overy, Heather Wilkinson

**Affiliations:** ^1^ Advanced Care Research Centre, University of Edinburgh, Edinburgh, United Kingdom; ^2^ Alzheimer Scotland Dementia Research Centre, University of Edinburgh, Edinburgh, United Kingdom; ^3^ Music in Human and Social Development, University of Edinburgh, Edinburgh, United Kingdom; ^4^ Edinburgh Centre for Research on the Experience of Dementia, University of Edinburgh, Edinburgh, United Kingdom; ^5^ Centre for Clinical Brain Sciences, University of Edinburgh, Edinburgh, United Kingdom; ^6^ Edinburgh Neuroscience, University of Edinburgh, Edinburgh, United Kingdom

## Abstract

**Background:**

Music activities are increasingly shown to be an effective nonpharmacological intervention to improve the lives of people living with dementia. For those living with dementia in care homes, music engagement is often facilitated through the work of an activities coordinator (AC). There is no specialised training to be an AC or standardised guidance in how ACs should approach their work, leaving ACs with a strong sense of autonomy but a lack of professional support. This study aims to explore the factors that influence how ACs in Scottish care homes plan and facilitate music activities, in order to improve guidelines and support for these professionals.

**Method:**

In this qualitative study, ten ACs from ten Scottish care homes were shadowed for up to twelve hours at work and took part in two in‐person interviews. The interviews were semi‐structured, with the questions informed by the shadowing visits. The transcribed interviews were analysed using reflexive thematic analysis.

**Result:**

Preliminary findings reveal a broad range of factors that can influence how ACs plan and facilitate music in care homes. These factors arise at the personal level (e.g. the ACs’ personal music facilitation confidence, sense of autonomy in their role, and knowledge of dementia care interventions), the contextual level (e.g. the cognitive abilities of residents, staff dynamics, and access to resources), and at the policy level (e.g. care inspectorate recommendations). Additionally, the findings highlight the diversity of experience for those within the AC role and the broader contextual diversity within long‐term‐care settings themselves.

**Conclusion:**

This research supports previous literature suggesting that ACs have a strong sense of autonomy in their role, while contributing new insights into how ACs make decisions regarding the planning and facilitation of music and other activities. It is hoped that the findings will inform practice for ACs aiming to engage meaningfully with music, help advise care home management and staff to better support ACs, and provide useful insights to external organisations seeking to collaborate with ACs. These potential changes to practice could improve the quality of music activities delivered by ACs, consequently enhancing the lives of those living in care homes.

